# Halogen-Bonded Supramolecular Parallelograms: From Self-Complementary Iodoalkyne Halogen-Bonded Dimers to 1:1 and 2:2 Iodoalkyne Halogen-Bonded Cocrystals

**DOI:** 10.1021/acs.cgd.3c01325

**Published:** 2024-02-09

**Authors:** Eric Bosch, Erin Speetzen, Nathan P. Bowling

**Affiliations:** † Department of Chemistry and Biochemistry, Missouri State University, 901 South National Avenue, Springfield, Missouri 65897, United States; ‡ Department of Chemistry, University of Wisconsin-Stevens Point, 2101 Fourth Avenue, Stevens Point, Wisconsin 54481, United States

## Abstract

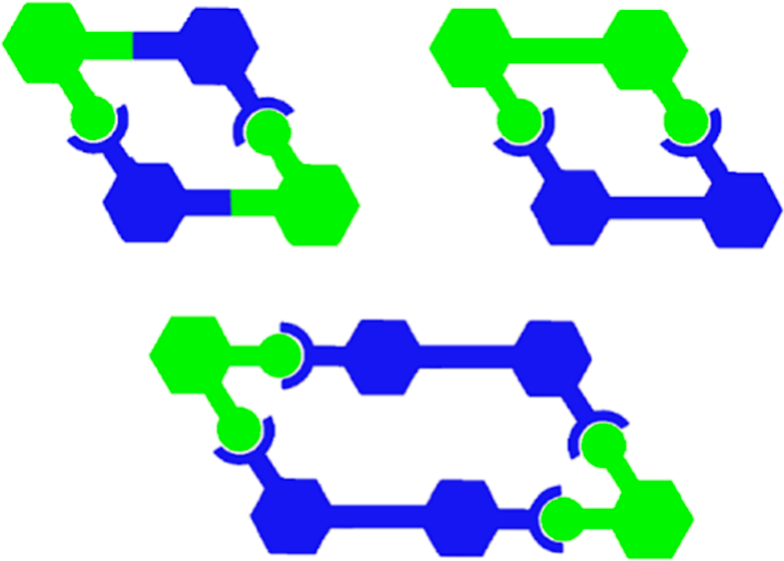

The formation of supramolecular parallelograms utilizing iodoalkyne–pyridine halogen bonding is described. The crystal structures of four iodoalkynyl-substituted (phenylethynyl)pyridines demonstrate the feasibility of discrete self-complementary dimer formation. These compounds 3-(2-iodoethynyl-phenylethynyl) pyridine (**1**), 2-(3-iodoethynyl-phenylethynyl) pyridine (**2**), 3-(4,5-difluoro-2-iodoethynyl-phenylethynyl) pyridine (**3**), and 2-(5-iodoethynyl-2,4-dimethylphenylethynyl) pyridine (**4**) all form parallelogram-shaped dimers with two self-complementary short N–I halogen bonds. The potential formation of iodoalkynyl halogen-bonded supramolecular macrocycles is demonstrated by the formation of a discrete halogen-bonded parallelogram-shaped complex in the 1:1 cocrystal formed from the bis iodoalkyne, 1-iodoethynyl-2-(3-iodoethynyl-phenylethynyl)-4,5-dimethoxybenzene (**6**), and the dipyridyl, 5-phenyl-2-(pyridin-3-ylethynyl)pyridine (**7**). Furthermore, discrete supramolecular parallelograms form within the 2:2 cocrystal formed between 1,2-*bis*(iodoethynyl)-4,5-difluorobenzene and the dipyridyl 4-(3-pyridylethynyl) pyridine (**8**).

## Abstract 2

The formation of parallelogram-shaped macrocycles facilitated by iodoalkyne–pyridine halogen bonds is described. These include self-complementary iodoalkynyl pyridine dimers, a 1:1 dipyridylethyne/*bis*(iodoalkynyl)tolane cocrystal, and a 2:2 bipyridylalkyne/*bis*(iodoalkynyl)benzene cocrystal.

## 1 Introduction

**Figure 1:**
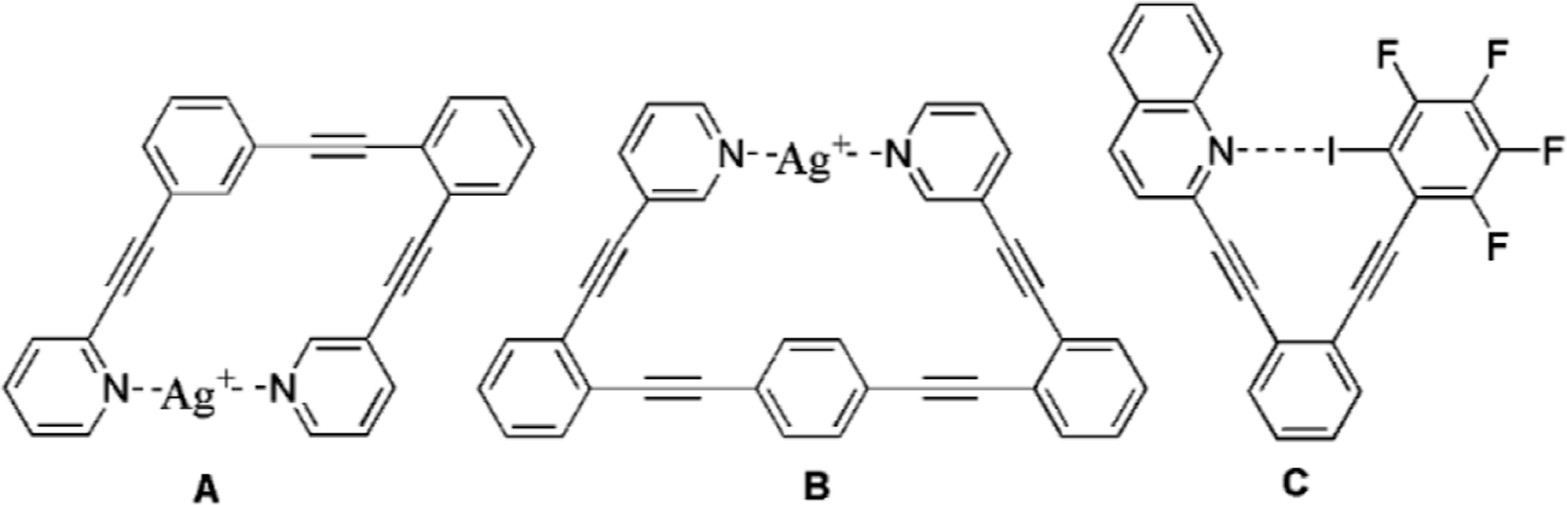
Planar π-conjugated arylethynylene systems: (A) rhomboidal coordination complex; (B) isosceles trapezoidal coordination complex; and (C) triangular halogen-bonded complex.

Synthetic macrocycles, including supramolecular macrocycles, have a wide variety of practical and potential applications. The broad field of host–guest chemistry^1^ encompasses many examples, including the precise preparation of porous materials for molecular separation^2^ and molecular identification via vapochromism.^3^ Applications in drug discovery have burgeoned over the past two decades, including specifically designed synthetic macrocycles.^4,5^ Here, we continue our interest in the development of π-conjugated arylethynylene macrocyles.^6^ We initially focused on the formation of simple planar macrocyclic coordination complexes from suitable conjugated bipyridyls. The planar triangular coordination complexes formed with the *trans*-coordinating ligand 1,2-*bis*(2-pyridylethynyl) benzene served as initial inspiration.^7,8^ We expanded the use of metal coordination to lock in coplanar macrocycle conformations with multiaryl systems forming rhomboidal^9^ (**A** in Figure 1), isosceles trapezoidal^10^ (**B** in Figure 1), and hexagonal π-conjugated coordination complexes.^11^

We concurrently extended these studies to halogen bonding.^12,13^ Our early halogen-bonding studies took advantage of the report that fluorine substitution increased the halogen-bonding strength of iodobenzene^14^ and lead us to investigate structures related to **C** in Figure 1. Indeed, these examples demonstrated the first intramolecular halogen-bonding-driven formation of a planar cyclic π-conjugated system.^15^ Halogen-bonded triangular trimolecular assemblies have subsequently been reported with 2-(iodoethynyl)pyridine and related compounds.^16,17^ We also demonstrated the formation of halogen-bonded parallelogram-shaped self-complementary dimers from appropriately oriented pyridyl-substituted polyfluoro iodobenzenes.^18,19^

In this study, we focus on iodoalkynes that are excellent halogen bond donors,^20,21^ with the caveat that they are, however, occasionally somewhat more fugitive during preparation.^22^ Accordingly, we report here the formation of parallelogram-shaped dimers featuring iodoalkynes as the halogen bond donor and further extend this to the intentional formation of 1:1 and 2:2 cocrystals that feature parallelogram-shaped supramolecular macrocycles using deliberate combinations of *bis*-iodoalkynes and bipyridines. This will complement the formation of halogen-bonded supramolecular rectangles on cocrystallization of 1,8-*bis*(iodoethynyl)anthracene with a variety of linear ditopic halogen bond acceptors.^23^ The formation of halogen-bonded supramolecular hexagons has been achieved using bipyridyl complexes of iodonium ions.^24^ Also, directly relevant to this study is the recent report of a parallelogram-shaped metallomacrocycle.^25^

## 2 Results and Discussion

### 2.1 Self-Complementary Dimer Formation

#### 2.1.1 Synthesis

**Figure 2:**
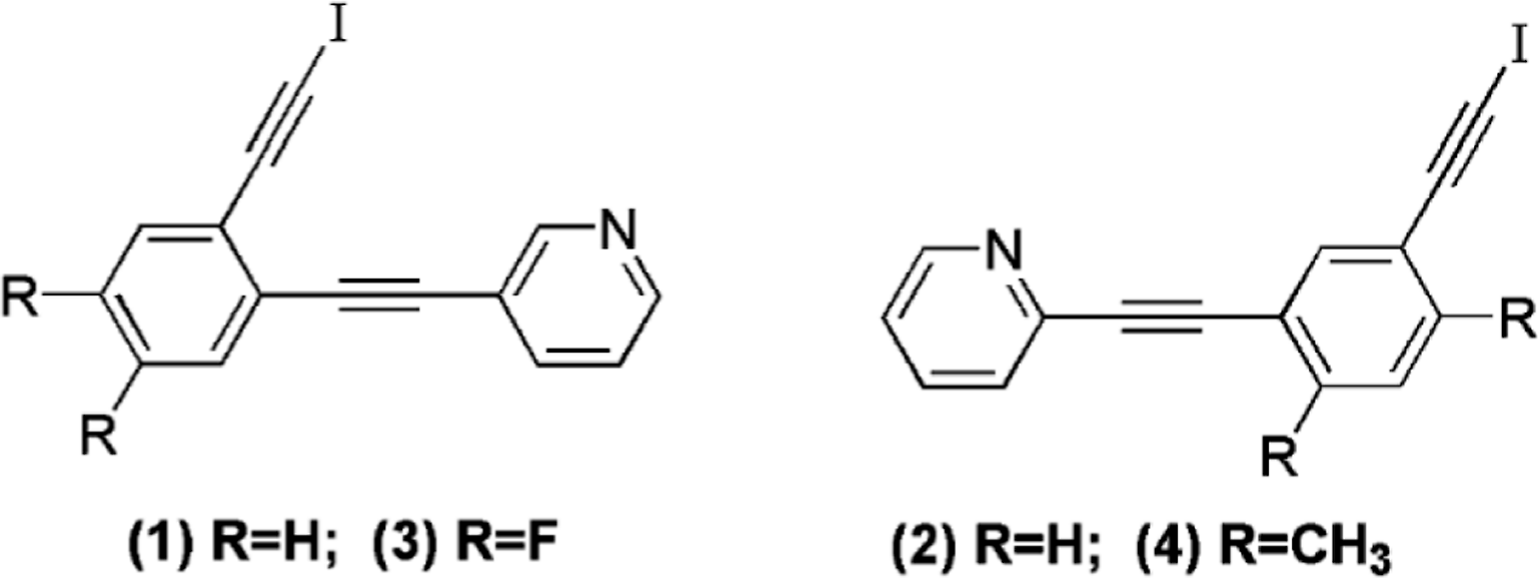
Iodoethynylpyridines were designed to form self-complementary dimers.

The series of iodoalkynylpyridines **1**–**4** shown in Figure 2 were designed to form self-complementary halogen-bonded dimers by intentional orientation of the pyridyl N and the iodoalkyne in the same direction. Each of the compounds was prepared by successive Sonogashira coupling reactions, followed by iodination.

Thus, reaction of 1-bromo-2-iodobenzene with a slight excess of 3-ethynylpyridine yielded 3-[2-bromophenyl)ethynyl] pyridine in good yield.

**Figure 3:**
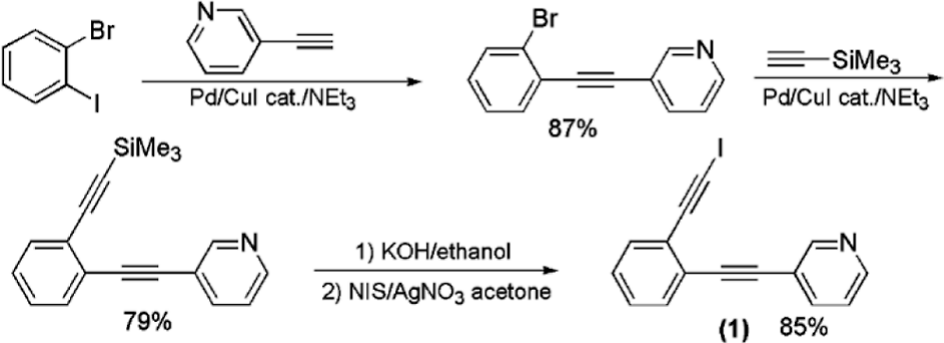
Synthesis of 3-[2-(2-bromophenyl) ethynyl] pyridine (**1**).

Subsequent palladium-catalyzed reaction with trimethylsilyl (TMS) acetylene and base-promoted deprotection yielded 3-[(2-ethynylphenyl) ethynyl] pyridine.^26^ The alkynylpyridine was then treated with NIS in acetone with a catalytic amount of silver(I) nitrate to give **1** as a colorless solid, as shown in Figure 3.^27^ Similar reaction of 1-bromo-3-iodobenzene with a slight excess of 2-ethynylpyridine yielded 2-[(3-bromophenyl) ethynyl] pyridine in good yield. Subsequent deprotection and iodination yielded **2** in a moderate yield.

Compounds **3** and **4** were prepared from 1,2-dibromo-4,5-difluorobenzene and 1,3-diiodo-4,6-dimethylbenzene, respectively, by reaction with less than one equivalent of the appropriate ethynylpyridine. Subsequent coupling of the monopyridyl product with TMS acetylene followed by deprotection and iodination yielded **3** and **4**, respectively, as colorless crystals. Each of the four compounds formed colorless crystals from chloroform on slow evaporation. It is noteworthy that the iodoalkynes feature a unique signal at around 10 ppm in the ^13^C NMR spectrum characteristic of sp-C bonded to the iodine atom.

#### 2.1.2 Crystallographic Analysis

**Figure 4:**
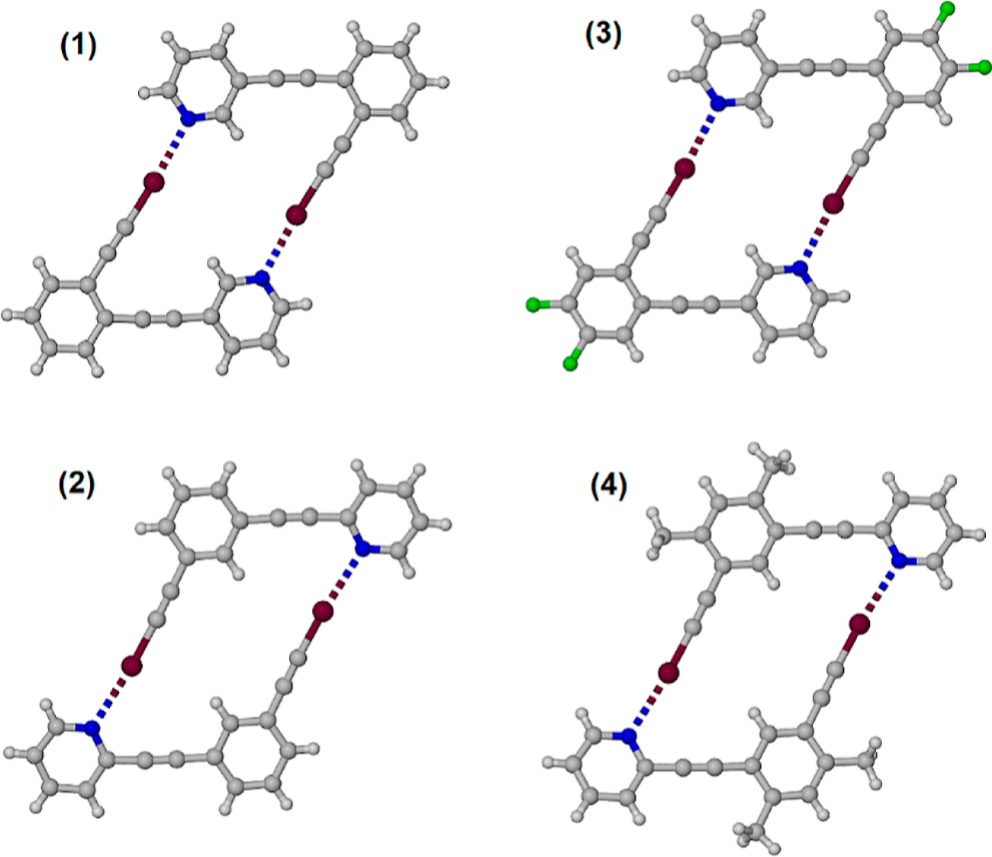
Self-complementary dimers corresponding to iodoethynylpyridines **1**–**4**. Atoms color coded with C atoms gray, N atoms blue, F atoms green, and I atoms maroon.

Each of the compounds **1**–**4** was crystallized from chloroform solution, and the single-crystal X-ray structures were determined at 100 K. The crystallographic data is collated in Table S1. Compound **1** crystallized in the monoclinic space group *P*21/*m* with one unique molecule in the asymmetric unit, forming a self-complementary sp–C–I···N halogen-bonded dimer (Figure 4). The halogen bond has an I–N separation of 2.773(16) Å, 79% of the sum of the van der Waals radii,^28^ with a C–I···N angle of 175.70(6)°. The halogen-bonded dimer is essentially planar, with a slight twist along the axis of the alkyne and an interplanar angle of 13.56(10)° between the two aromatic rings. The iodoalkyne moiety is slightly bent with the iodine atom 0.570 (4) Å above the plane of the benzene ring. Compounds **2–4** each form similar, essentially planar, self-complementary dimers, as shown in Figure 4.

Each dimer features a short iodine–nitrogen separation ranging from 2.77 to 2.97 Å, 78–84% of the sum of the van der Waals radii, and near-linear C–I···N angles of 175.5 to 176.5°, as collated in Table 1.

**Table 1:** Halogen Bond Distances and Angles for Self-Complementary Dimers **1–4**

compound	1	2	3	4
I···N dist., Å	2.769(2)	2.8424(19)	2.780(2)	2.967(3)
C–I···N ang., °	175.67(8)	175.55(8)	176.5(9)	175.78(11)

Given that the formation of these halogen-bonded parallelograms is part of a larger project aimed at forming π-stacked macrocycles with internal and extended void space, we subjected each structure to the void space probe, 1.2 Å probe radius, using the program Mercury.^29^ In each case, there was 0% void space.

#### 2.1.3 Hirshfeld Surface Analysis, Intermolecular Interaction Energy Analysis, and Molecular Electrostatic Potentials

**Figure 5:**
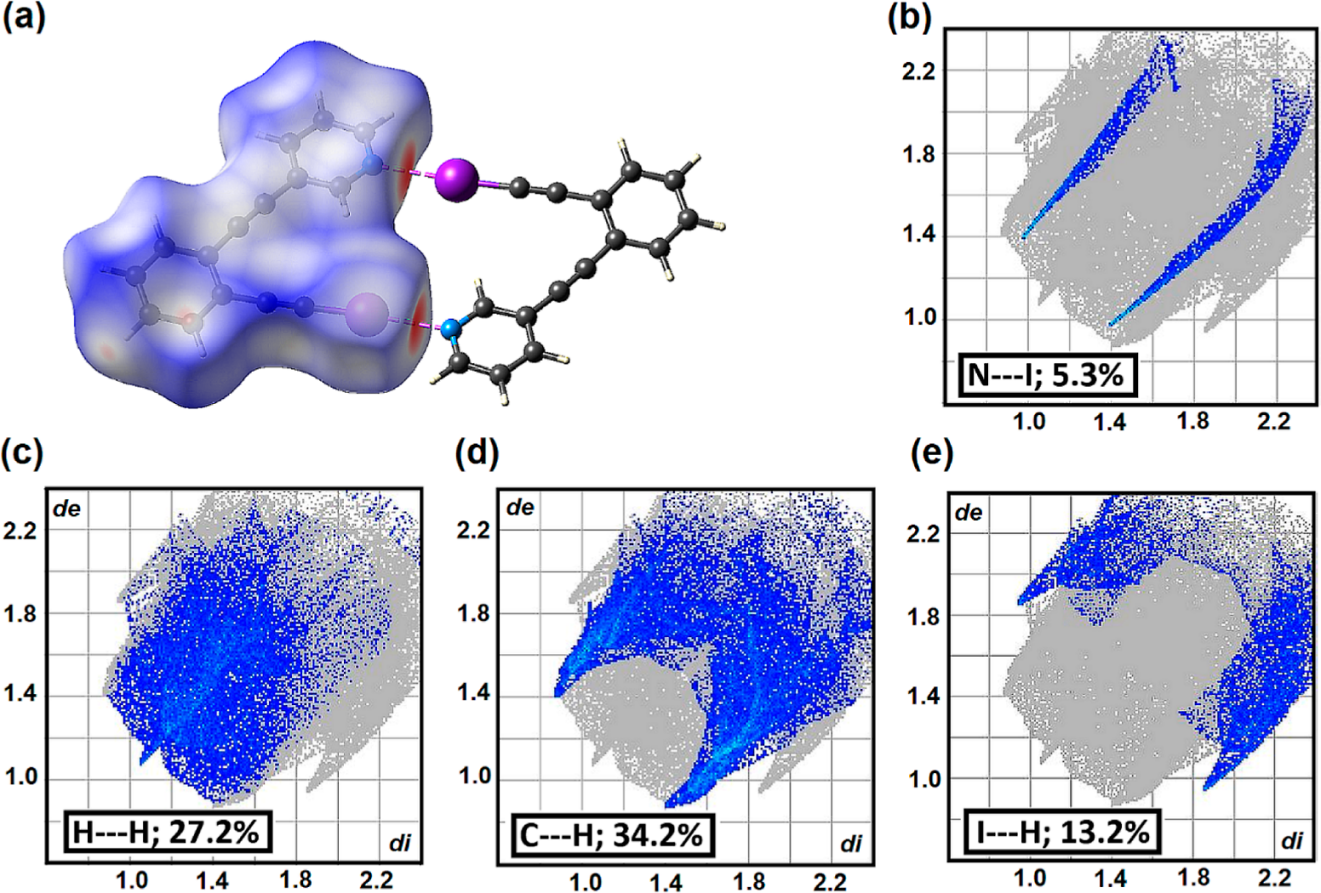
(a) Hirshfeld surface of **1** mapped over *d*_norm_. Short and long contacts are indicated as red and blue regions, respectively. The molecule that forms the halogen-bonded dimer is shown with the halogen bonds represented by red dashed lines. In all cases, reciprocal contacts are included. The two-dimensional fingerprint plots, along with the relative percentages, for **1** are delineated into (b) N···I contacts, (c) H···H contacts, (d) C···H contacts, and (e) I···H contacts.

The N···I close contacts in Table 1 and other contacts within crystals **1**–**4** were analyzed by generation and analysis of the Hirshfeld surface using CrystalExplorer.^30,31^ In these plots, short and long contacts, relative to the respective van der Waals radii, are indicated as red and blue regions, respectively. In each case, reciprocal contacts are included. Thus, N···I includes contacts in which the N atom is within the Hirshfeld surface and the I atom outside, as well as contacts in which the I atom is within the Hirshfeld surface and the N atom outside, I···N. In Figure 5a, the N···I contacts are represented by the deepest red coloration on the surface. The atom-to-atom contacts filtered by element, known as fingerprint plots, reveal that the N···I contacts correspond to only 5.3% of the surface area of the central molecule (Figure 5b). Indeed, the surface contacts are predominantly C···H (34.2%), H···H (27.2%), and I···H (13.2%). These contributions are plotted in Figure 5c–e. The complete element-by-element breakdown of the contacts for **1–4** is collated in Table S2. While the Hirshfeld surface contacts filtered by element are similar for **1** and **2**, compound **3** has two F atoms and two fewer H atoms than **1**. Consequently, there are significant surface contacts between F and H and between F and C. Similarly, since **4** has two methyl groups, there are increased H–H contacts relative to **1**.

**Figure 6:**
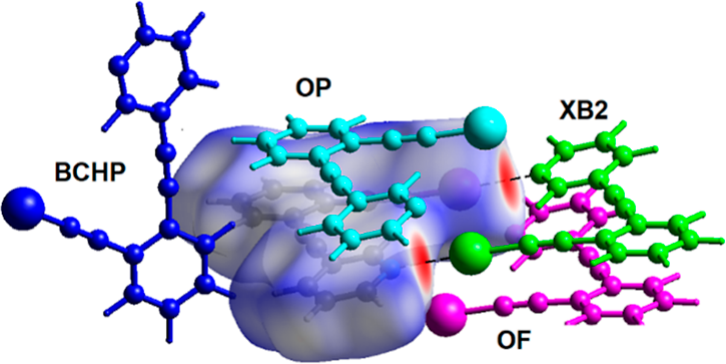
Hirshfeld surface for molecule **(1)**, with *d*_norm_ mapped over the surface with the close contacts colored red. The four molecules within 3.8 Å that have the strongest energy of interaction with the central molecule are shown color coded. Green, XB2 = halogen-bonded; light blue, OP = offset π-stacked; pink, OF = offset; and dark blue, BCHP = bifurcated C–H···alkyne. The I···N halogen bond is shown as dashed lines.

The intermolecular energy of interaction between a central molecule **(1)** and molecules within 3.8 Å was also calculated using CrystalExplorer.^32,33^ These calculations revealed that the halogen-bonded molecule, green XB2 in Figure 6, has the strongest molecule–molecule interaction with an *E*_tot_ of −56.2 kJ/mol. The offset π-stacked molecule, light blue OP in Figure 6, has *E*_tot_ = −38.1 kJ/mol, while the remaining molecules in close contact have significantly lower energies of interaction. Specifically, −18.8 kJ/mol for the molecule with a bifurcated C–H···π interaction, dark blue BCHP in Figure 6, and −16.4 kJ/mol for the second offset molecule, pink OF in Figure 6. The full data are collated in Table S3. Similar calculations with **2** and **3** revealed that the strongest intermolecular interaction between the molecules within the crystal was to the halogen-bonded partner with total energies of interaction, *E*_tot_, of −62.1 and −57.4 kJ/mol, respectively. This data are collated in Tables S4 and S5, with accompanying Figures S1 and S2, respectively.

**Figure 7:**
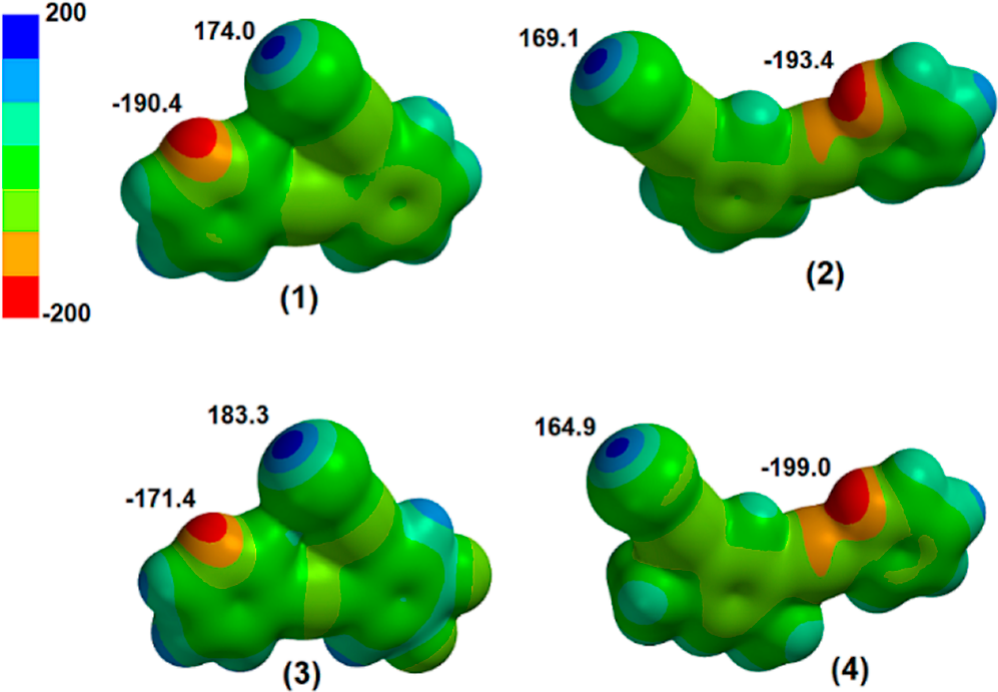
Calculated molecular electrostatic potential for molecules **(1)** to **(4)** is shown with the same scale corresponding to the color-coded legend (−200 to +200 kJ mol^–1^) with minima and maxima annotated on each surface.

The relative molecular electrostatic potentials of the molecules **(1)** to **(4)** were determined using Spartan’20.^34^ The maximum and minimum molecular electrostatic potentials listed in Table 2 correspond to the planar conformations observed in the crystal structures. In each case, the maximum electrostatic potential or σ-hole was observed on the iodine atom along the C–I axis, and the minimum electrostatic potential was located on the nitrogen atom corresponding to the location of the pyridyl nonbonding electron pair. The highest σ-hole in this group corresponded to **(3)**, and this is reasonably a consequence of the electron withdrawing effect of the two fluorine atoms on the benzene ring with a correspondingly lesser minimum molecular electrostatic potential on the pyridyl N. In line with the electron-donating effect of the two methyl substituents, **(4)** has the least positive σ-hole and the most negative potential on the pyridyl N. This substituent effect is visible in the electrostatic potential plots shown in Figure 7. For comparison, the σ-hole values for **(1)**–**(4)** are collated in Table 2. Note that these values are similar to the value of 174.6 kJ/mol calculated for iodoperfluorobenzene under identical conditions.

**Table 2:** Maximum and Minimum Electrostatic Potentials for Molecules **1–4** in kJ mol^–1^

compound	1	2	3	4
*V*_s_ (max)	174.0	169.1	183.3	164.9
*V*_s_ (min)	–190.4	–193.4	–171.4	–199.0

### 2.2 Cocrystal Formation

We reasoned that the strength of the cooperative iodoalkyne–pyridine interaction coupled with the relative ease of directional placement of substituents about pyridine and benzene rings provided a strategy to direct the formation of larger discrete macrocyclic halogen-bonded systems through cocrystallization. To test this hypothesis, we first prepared a *bis*(iodo)alkyne and a complementary bipyridyl designed to form a 1:1 cocrystal, featuring a parallelogram-shaped supramolecular macrocycle within the structure. A second couple comprising a *bis*-iodoalkyne and a bipyridyl were designed to form a 2:2 cocrystal, featuring a parallelogram-shaped supramolecular macrocycle. In each cocrystal, the individual components themselves are crystalline solids.

#### 2.2.1 1:1 Cocrystal Formation

**Figure 8:**
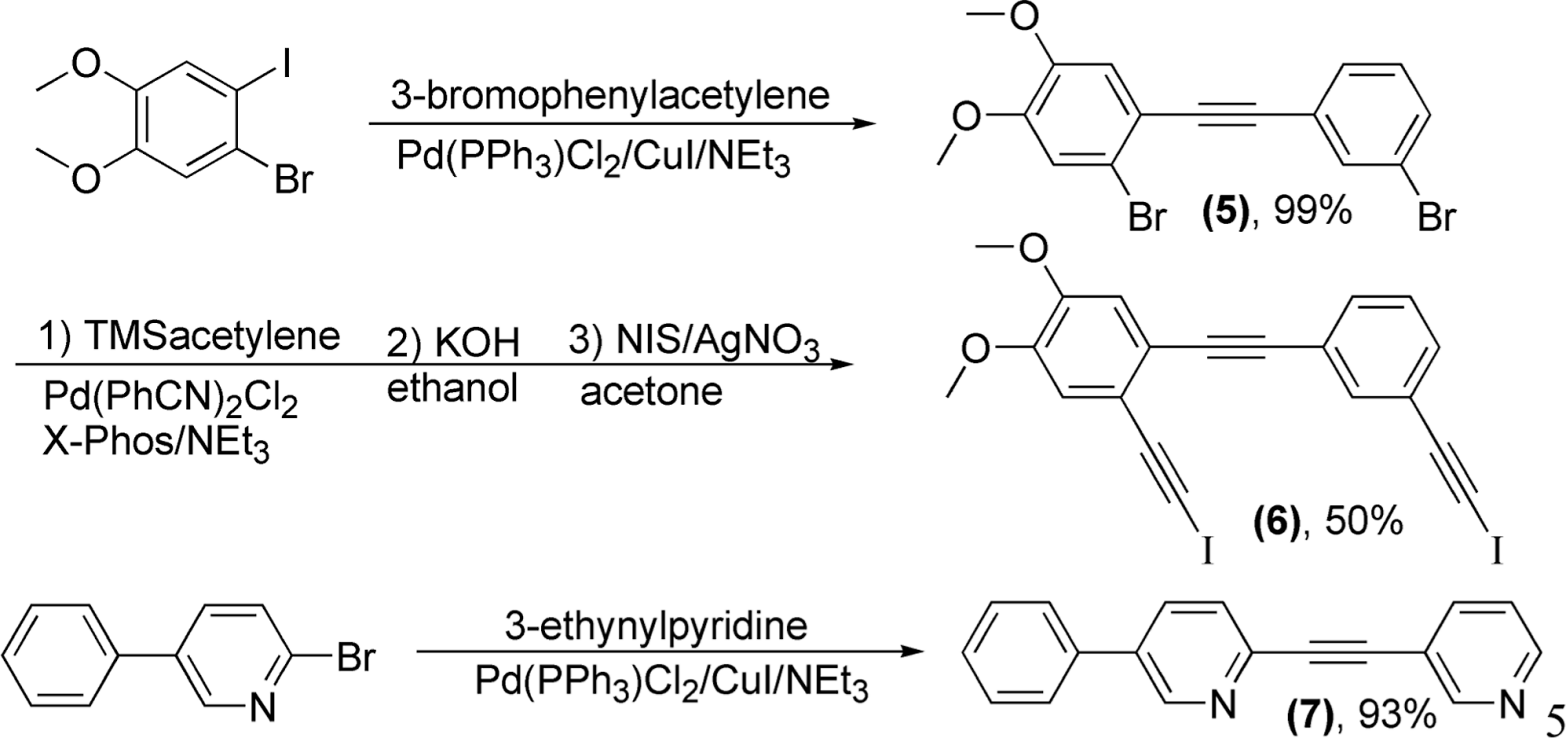
Synthesis of *bis*-iodoalkyne **(6)** and dipyridylethyne **(7)**.

Our planned 1:1 cocrystal strategy required that both components have rotational freedom but are able to adopt a conformation suitable for cooperative halogen bonding. To this end, *bis*-iodoethynyl derivative **(6)** was synthesized (Figure 8). To achieve this, 3-bromo-4-iodoveratrole was coupled with 3-bromophenylacetylene, yielding dibromodimethoxy tolane **(5)** in excellent yield.^35^ Tolane **(5)** was then coupled with excess TMS acetylene, followed by desilylation and iodination to form **(6)** in moderate yield over three steps. Dipyridyl **(7)** was formed by palladium-catalyzed coupling of 2-bromo-5-phenylpyridine with 3-ethynylpyridine. Both **(6)** and **(7)** have a second planar conformation, as compared to the conformation shown in Figure 8, in which the halogen-bonding moieties do not face in the same direction, rather facilitating the formation of an extended one-dimensional polymer.

**Figure 9:**
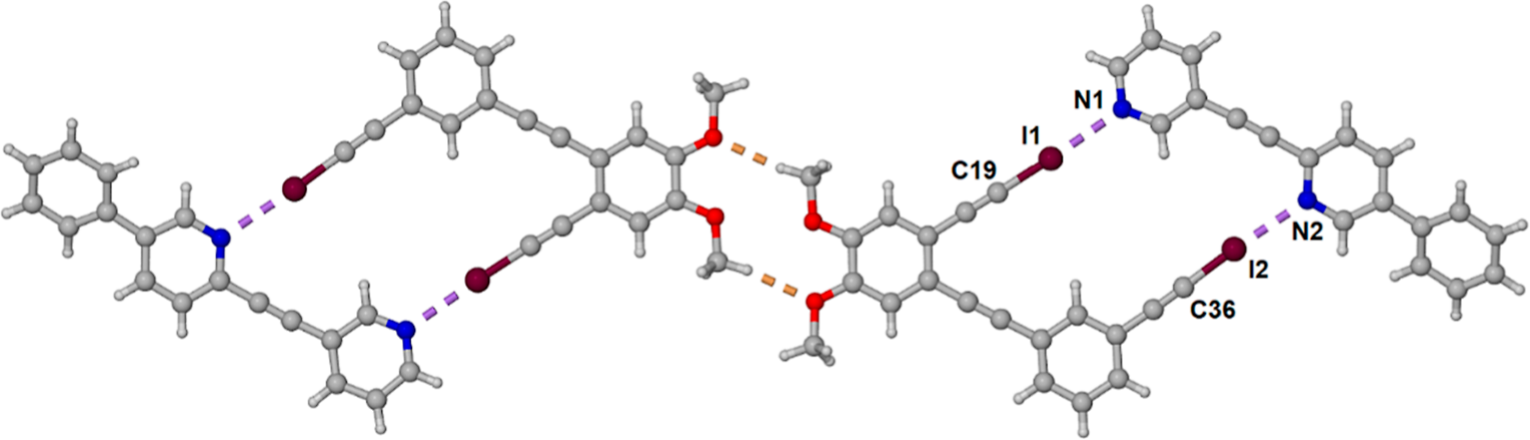
Asymmetric unit of the 1:1 cocrystal formed between bis iodoalkyne **(6)** and dipyridyl **(7)** along with a second asymmetric unit connected through self-complementary C–H···O hydrogen bonding. Atoms are color coded with C gray, N blue, O red, and I maroon. Halogen bonds are shown as purple dashed lines, and hydrogen bonds are shown with orange dashed lines.

The formation of the 1:1 cocrystal involved dissolving one equivalent of each of the components **(6)** and **(7)** in a 1:1 by volume mixture of ethyl acetate and dichloromethane. The solvent was allowed to slowly evaporate resulting in the formation of gold colored block-shaped crystals. Analysis by single-crystal X-ray diffraction revealed that the cocrystal crystallized in the monoclinic space group *P*2_1_/*n* with one molecule of each of the components **(6)** and **(7)** in the asymmetric unit (Figure 9). The core tetraarylethynylene moiety is essentially planar, although the dipyridyl alkyne is slightly bent and the attached external phenyl ring is twisted with respect to the attached pyridine with a torsional angle of 29.86(11)°. The I···N separation distances of 2.804(3) and 2.823(3) Å for I1···N1 and I2···N2 are 79 and 80% of the sum of the van der Waals radii with near linear C–I···N angles of 178.00(11) and 174.85(12)°, respectively. Adjacent parallelograms interact through self-complementary C–H···O hydrogen bonding that we had earlier established as a relatively common interaction in crystal structures containing the 1,2-dimethoxy moiety.^36^

Within the unit cell, the hydrogen-bonded couples of parallelogram-shaped **6**–**7** units shown in Figure 9 do not π-stack. Nevertheless, in this structure, a void space of 53 Å^3^ was revealed with a 1.2 Å probe radius. The space is, however, isolated and divided into three locations and therefore essentially inaccessible to solvents or other small molecules (Figure S3).

#### 2.2.2 2:2 Cocrystal Formation

**Figure 10:**
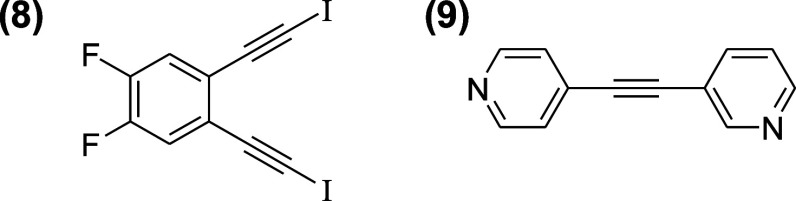
Components designed and synthesized for 2 + 2 cocrystallization.

We further reasoned that 2:2 cocrystallization of a 1,2-*bis*(iodoethynyl) benzene and the bipyridyl, 3-(4-pyridylethynyl) pyridine might lead to a parallelogram-shaped halogen-bonded core supramolecular macrocycle with the *bis*-iodoalkyne at each acute corner. To this end, coupling 1,2-dibromo-4,5-difluorobenzene with excess TMS acetylene followed by deprotection yielded 1,2-diethynyl-4,5-difluorobenzene^37^ that was iodinated to form 1,2-*bis*(iodoethynyl)-4,5-difluorobenzene **(8)**. Separately, 4-iodopyridine was coupled with 3-ethynylpyridine to provide the complementary component for 2 + 2 cocrystallization, namely, 3-(4-pyridylethynyl) pyridine **(9)**,^38^ as shown in Figure 10.

**Figure 11:**
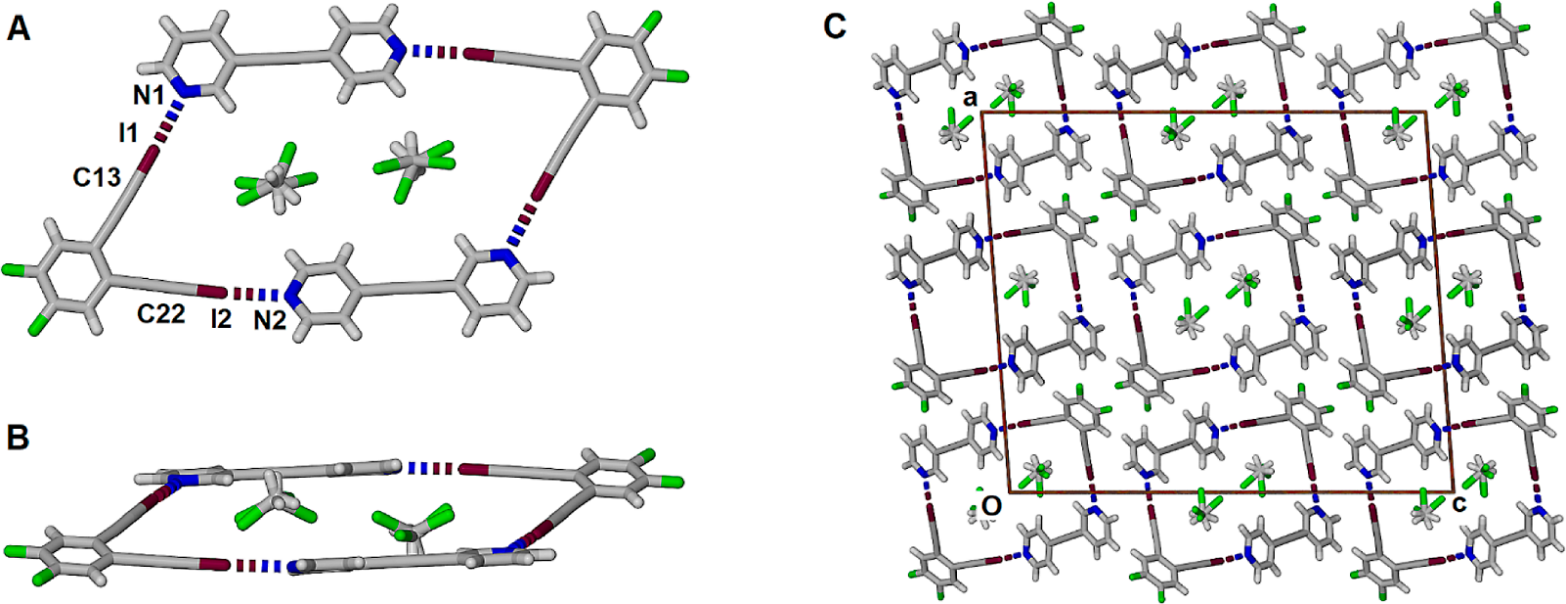
(A) Parallelogram-shaped macrocycle formed within the 2:2 cocrystal formed between **(8)** and **(9)**, showing disordered dichloromethane. (B) View along the plane of the bipyridyls in a discrete parallelogram. (C) Packing is shown along the *b* axis. Atoms color coded with C atoms gray, N atoms blue, F atoms green, and I atoms maroon.

Slow evaporation from a dichloromethane solution containing equimolar amounts of **(8)** and **(9)** yielded small golden block-shaped crystals. The cocrystal crystallized in the monoclinic space group *I*2/*a*, and the asymmetric unit contained one molecule of each of **(8)** and **(9)**, along with a disordered dichloromethane molecule. The molecules are indeed set up as a 2:2 cocrystal, forming a parallelogram with 4 halogen bonds binding the four components, as shown in Figure 11A. The two unique halogen bonds have I–N separations of 2.733(4) and 2.776(5) Å for I1···N1 and I2···N2 that are 77 and 79% of the sum of the van der Waals radii with near linear C–I···N angles of 174.28(19) and 176.57(18)°, respectively. The aromatic rings are not coplanar but are offset, as shown in the side-view in the plane of the bipyridyl (Figure 11B). The dichloromethane molecule is disordered over two major positions in a ratio of 3:2. The packing in Figure 11C shows the stacking of adjacent columns of parallelograms to form channels that contain pairs of disordered dichloromethane molecules parallel to the *b* axis. There is a type I Cl···Cl interaction between chlorines of the major component of the disordered dichloromethane with a Cl···Cl separation of 2.770 Å (77% of the sum of the van der Waals radii) and a C–Cl···Cl angle of 150.16°. In order to evaluate this interaction, a search of the Cambridge Database^39^ for similar symmetric type I interactions between dichloromethane molecules was undertaken (see Figure S4 for search criteria). The average and median Cl···Cl separations for the 125 distinct instances are 3.217 and 3.277 Å, respectively. Of the 125 distinct instances with Cl···Cl separations within the range of 2.6 to 3.4 Å, 12 of the 14 shorter distances, all less than 3.000 Å, involved disordered dichloromethane molecules. Nonetheless, a type I interaction with a Cl···Cl separation of 2.669 Å between dichloromethane molecules (without disorder) was found in the structure of the *bis*(6-bromo-1,2-dihydroacenaphthylen-5-yl)(ethyl)arsane dichloromethane solvate.^40^ The separation we report here is close enough to suggest that the major component of the disorder is preferentially paired with the minor component, thereby mostly avoiding close contact. No additional solvent-accessible void spaces were detected using a 1.2 probe radius.

## 3 Experimental Section

### 3.1 Synthesis

#### 3.1.1 Synthesis of 3-(2-Iodoethynyl-phenylethynyl)pyridine **(1)**

The precursor for iodoalkyne (**1**) was prepared from 1-bromo-2-iodobenzene by sequential coupling reactions. Thus, palladium-catalyzed coupling of 1-bromo-2-iodobenzene with 3-ethynylpyridine yielded 3-[(2-bromophenyl)ethynyl]pyridine that was then coupled with TMS acetylene, followed by base hydrolysis to yield 3-[(2-ethynylphenyl)ethynyl]pyridine with spectral data identical to that previously reported.^26^

A solution of 3-[(2-ethynylphenyl)ethynyl]pyridine (0.160 g, 0.79 mmol) in acetone (5 mL) under argon was treated with *N*-iodosuccinimide (0.249 g, 1.11 mmol) and silver(I) nitrate (0.034 g, 0.20 mmol). The reaction was sealed and stirred for 24 h. The reaction crude was extracted with ethyl acetate, and the extract was washed with water and brine and dried over sodium sulfate. After evaporation of the solvent, the product was purified by flash chromatography to yield the product as colorless crystals (0.227 g, 85%). ^1^H NMR (400 MHz, CDCl_3_): δ 8.82 (br d, *J* = 2.0 Hz, 1H), 8.56 (dd, *J* = 1.6, 7.8 Hz, 1H), 7.85 (td, *J* = 2.0, 7.6 Hz, 1H), 7.54–7.52 (m, 1H), 7.49–7.47 (m, 1H), 7.34–7.29 (m, 3H). ^13^C NMR (100 MHz, CDCl_3_): δ 152.5, 148.7, 138.6, 132.5, 131.5, 128.6, 128.4, 126.2, 123.1, 120.3, 92.7, 91.1, 90.2, 11.5.

#### 3.1.2 Synthesis of 2-(3-Iodoethynyl-phenylethynyl)pyridine **(2)**

2-[(3-Ethynylphenyl)ethynyl]pyridine was synthesized from 1-bromo-3-iodobenzene and 2-ethynylpyridine prepared as described for 3-[(2-ethynylphenyl)ethynyl]pyridine above. Treatment of 2-(3-ethynylphenyl)ethynyl)pyridine (0.295 g, 1.45 mmol) with NIS and silver(I) nitrate in acetone as described for **(1)** yielded 2-(3-iodoethynyl-phenylethynyl) pyridine as a colorless solid (0.393 g, 82%). ^1^H NMR (400 MHz, CDCl_3_): δ 8.62 (md, *J* = 4 Hz, 1H), 7.71–7.66 (m, 2H), 7.55 (td, *J* = 1.2, 7.6 Hz, 1H), 7.52 (d, *J* = 7.6 Hz, 1H), 7.42 (td, *J* = 1.2, 7.6 Hz, 1H), 7.31 (t, *J* = 7.6 Hz, 1H), 7.27–7.24 (m, 1H). ^13^C NMR (100 MHz, CDCl_3_): δ 150.3, 143.3, 136.4, 135.8, 132.9, 132.4, 128.6, 127.5, 124.0, 123.2, 122.8, 93.2, 89.4, 88.3, 8.3.

#### 3.1.3 Synthesis of 4,5-Difluoro-1-(iodoethynyl)-2-(3-pyridylethynyl)benzene **(3)**

1,2-Diethynyl-4,5-difluorobenzene was synthesized by palladium-catalyzed 1,2-dibromo-4,5-difluorobenzene with excess TMS acetylene, followed by base-catalyzed deprotection.^37^ Argon was bubbled through a solution of 1,2-diethynyl-4,5-difluorobenzene (0.840 g, 6.1 mmol) and 3-iodopyridine (0.685 g, 3.3 mmol) in triethylamine (20 mL) for 5 min before *bis*(triphenylphosphine)palladium(II) chloride (0.074 g) and copper(I) iodide (0.034 g) were added, and the flask was sealed and stirred at room temperature for 72 h. The products were separated by flash chromatography to yield 3,4-difluoro-1-(ethynyl)-6-(3-pyridylethynyl) benzene as a pale orange solid (0.482 g, 65%). ^1^H NMR (400 MHz, CDCl_3_): δ 8.79 (s, 1H), 8.58 (d, *J* = 4.0 Hz, 1H), 7.82 (td, *J* = 1.8, 8.0 Hz, 1H), 7.37–7.29 (m, 3H), 3.39 (s, 1H). Treatment of 4,5-difluoro-1-(ethynyl)-2-(3-pyridylethynyl)benzene with *N*-iodosuccinimide and silver(I) nitrate in acetone as before gave **(3)** in 63% yield. ^1^H NMR (400 MHz, CDCl_3_): δ 8.80 (d, *J* = 1.6 Hz, 1H), 8.58 (dd, *J* = 1.6, 5.2 Hz, 1H), 7.82 (td, *J* = 1.8, 6.0 Hz, 1H), 7.33–7.24 (m, 3H). ^13^C NMR (100 MHz, CDCl_3_): δ 152.6, 150.3 (ddd, *J* = 256, 32, 5 Hz), 149.2, 138.6, 123.5 (ddd, *J* = 18.6 Hz), 123.1, 121.5 (br d, *J* = 72 Hz), 120.4 (dd, *J* = 12, 72 Hz), 119.9, 91.0 (m), 89.2 (m), 13.1. ^19^F NMR (376 MHz, CDCl_3_): δ 134.2 (ddd, *J* = 21.4, 10.5, 7.9 Hz, 1F), 134.0 (ddd, *J* = 21.4, 10.5, 7.9 Hz, 1F).

#### 3.1.4 Synthesis of 2-(5-Iodoethynyl-2,4-dimethyl-phenylethynyl)-pyridine **(4)**

4,6-Diiodo-*m*-xylene was coupled with 0.5 equiv of 2-ethynylpyridine with palladium catalyst as described before. After TLC indicated the absence of 2-ethynylpyridine, an excess of TMS acetylene was added. The monopyridyl product 2-(2,4-dimethyl-5-trimethylsilanylethynyl-phenylethynyl)-pyridine was isolated in a moderate yield (56%). ^1^H NMR (400 MHz, CDCl_3_): δ 8.62 (ddd, *J* = 1.0, 2.0, 4.9 Hz, 1H), 7.67 (dt, *J* = 1.9, 7.8 Hz, 1H), 7.65 (s, 1H), 7.50 (md, *J* = 8 Hz, 1H), 7.23 (ddd, *J* = 1.5, 4.9, 7.8 Hz, 1H), 2.50 (s, 3H), 2.41 (s, 3H), 0.26 (s, 9H). This was deprotected to yield 2-(5-ethynyl-2,4-dimethyl-phenylethynyl)-pyridine in quantitative yield ^1^H NMR (400 MHz, CDCl_3_): δ 8.80 (ddd, *J* = 1.5, 5.2, 5.2 Hz, 1H), 8.58 (dd, *J* = 1.6, 5.2 Hz, 1H), 7.70–7.65 (m, 2H), 7.51 (dd, *J* = 1.2, 6.8 Hz, 1H), 7.24 (ddd, *J* = 1.2, 5.0, 7.4 Hz, 1H), 3.25 (s, 1H), 2.51 (s, 3H), 2.43 (s, 3H). ^13^C NMR (100 MHz, CDCl_3_): δ 150.3, 143.8, 141.9, 141.6, 136.4, 136.3, 131.1, 127.4, 122.9, 119.9, 92.5, 87.5, 81.8, 81.0, 20.9, 20.8. The ethynylpyridine derivative was iodinated with NIS with catalytic quantities of silver(I) nitrate in acetone as described before to form 2-(5-iodoethynyl-2,4-dimethyl-phenylethynyl)-pyridine in moderate yield (37%). ^1^H NMR (400 MHz, CDCl_3_): δ 8.63 (br d, *J* = 4.4 Hz, 1H), 7.68 (dt, *J* = 1.8, 7.6 Hz, 1H), 7.60 (s, 21H), 7.51 (d, *J* = 7.6 Hz, 1H), 7.26–7.22 (m, 1H), 7.08 (s, 1H), 2.51 (s, 3H), 2.42 (s, 3H). ^13^C NMR (100 MHz, CDCl_3_): δ 150.1, 143.6, 142.1, 141.4, 136.4, 136.1, 130.8, 127.2, 122.7, 120.6, 119.6, 92.32, 92.27, 87.3, 20.7, 20.6, 9.0.

#### 3.1.5 Synthesis of 1-Iodoethynyl-2-(3-iodoethynyl-phenylethynyl)-4,5-dimethoxy-benzene **(6)**

Palladium-catalyzed coupling of 4-bromo-5-iodo-1,2-dimethoxybenzene (1.01 g) with 1 equiv of 3-bromophenylacetylene yielded 1-bromo-2-(3-bromo-phenylethynyl)-4,5-dimethoxy-benzene in excellent yield (1.12 g, 99%). ^1^H NMR (400 MHz, CDCl_3_): δ 7.71 (t, *J* = 1.8 Hz, 1H), 7.49–7.45 (m, 2H), 7.22 (t, *J* = 8.0 Hz, 1H), 7.06 (s, 1H), 7.01 (s, 1H), 3.89 (s, 3H), 3.88(s, 3H). ^13^C NMR (100 MHz, CDCl_3_): δ 150.1, 148.1, 134.2, 131.5, 130.1, 129.8, 125.1, 122.2, 117.1, 116.6, 115.2, 115.0, 90.7, 89.5, 56.2, 56.1. This product was then treated with excess TMS acetylene to yield the *bis*(trimethylsilyl) derivative in good yield (0.983 g, 81%). This was immediately deprotected with sodium hydroxide in ethanol to give 1-ethynyl-2-(3-ethynyl-phenylethynyl)-4,5-dimethoxy-benzene (0.476 g, 62%). ^1^H NMR (400 MHz, CDCl_3_): δ 7.68 (t, *J* = 1.8 Hz, 1H), 7.52 (td, *J* = 1.5, 8.0 Hz, 1H), 7.39 (td, *J* = 1.5, 8.0 Hz, 1H), 7.31 (t, *J* = 7.6 Hz, 1H), 6.95 (s, 1H), 6.93 (s, 1H), 3.91 (s, 3H), 3.89 (s, 3H). ^13^C NMR (100 MHz, CDCl_3_): δ 149.4, 149.1, 135.1, 131.9, 131.8, 128.4, 123.7, 122.4, 119.0, 117.7, 114.7, 114.0, 91.1, 88.7, 82.8, 82.2, 79.9, 77.8, 56.03, 56.02. Treatment of 1-ethynyl-2-(3-ethynyl-phenylethynyl)-4,5-dimethoxy-benzene (0.485 g) with iodine and sodium hydroxide in methanol, as described by Aakeroy et al.,^23^ yielded a precipitate of 1-iodoethynyl-2-(3-iodoethynyl-phenylethynyl)-4,5-dimethoxy-benzene as an off-white solid (0.628 g, 71%). ^1^H NMR (400 MHz, CDCl_3_): δ 7.63 (t, *J* = 1.6 Hz, 1H), 7.50 (td, *J* = 1.4, 7.6 Hz, 1H), 7.44 (td, *J* = 1.4, 7.6 Hz, 1H), 7.30 (t, *J* = 7.6 Hz, 1H), 6.989 (s, 1H), 6.985 (s, 1H), 3.91 (s, 3H), 3.89 (s, 3H), 3.32 (s, 1H), 3.10 (s, 1H). ^13^C NMR (100 MHz, CDCl_3_): δ 149.4, 149.0, 135.4, 132.0, 131.9, 128.4, 123.7, 123.6, 119.7, 119.2, 114.6, 113.6, 93.4, 92.9, 91.4, 88.8, 79.9, 77.8, 56.03, 8.8, 7.2.

#### 3.1.6 Synthesis of 2-(Pyridineethynyl)-5-phenylpyridine (**7**)

Palladium-catalyzed coupling of 2-bromo-5-phenylpyridine (0.371 g, 0.16 mmol) with 1.05 equiv of 3-ethynylpyridine yielded the title compound in good yield as a colorless solid (0.382 g, 93%). ^1^H NMR (400 MHz, CDCl_3_): δ 8.88 (d, *J* = 2.4 Hz, 1H), 8.85 (d, *J* = 2.0 Hz, 1H), 8.60 (dd, *J* = 2.0, 5.0 Hz, 1H), 7.764–7.60 (m, 2H), 7.51 (mt, *J* = 8.0 Hz, 2H), 7.32 (dd, *J* = 5.0, 7.6 Hz, 1H). ^13^C NMR (100 MHz, CDCl_3_): δ 152.8, 149.4, 148.9, 141.6, 139.1, 137.2, 136.3, 134.6, 129.4, 128.8, 127.4, 127.3, 123.3, 119.8, 91.9, 86.4.

#### 3.1.7 Synthesis of 1,2-*Bis*(iodoethynyl)-4,5-difluorobenzene **(8)**

Palladium-catalyzed coupling of 1,2-dibromo-4,5-difluorobenzene with excess TMS acetylene followed by base-promoted deprotection yielded 1,2-diethynyl-4,5-difluoro-benzene in good yield.^37^ Iodination, as described for **(6)**, yielded the title compound as an orange solid (0.107 g, 49%). ^1^H NMR (400 MHz, CDCl_3_): δ 7.26–7.18 (m, 2H). ^13^C NMR (100 MHz, CDCl_3_): δ 150.0 (ddd, *J* = 253.4, 28.0, 12.7 Hz), 121.5 (dd, *J* = 2.4, 17.2 Hz), 121.2 (dd, *J* = 5.4, 15.3 Hz), 82.0 (d, *J* = 1.5 Hz), 12.7. ^19^F NMR (376 MHz, CDCl_3_): δ 133.8.

#### 3.1.8 Synthesis of 3-(4-Pyridineethynyl)pyridine **(9)**

Palladium-catalyzed coupling of 3-ethynylpyridine with 4-iodopyridine yielded the title compound as a colorless solid (0.165 g, 91%).^38^^1^H NMR (400 MHz, CDCl_3_): δ 8.80 (t, *J* = 1.0 Hz, 1H), 8.65–8.63 (td, *J* = 1.4, 4.4 Hz, 2H), 8.61 (td, *J* = 1.5, 4.4 Hz, 1H), 7.84 (qd, *J* = 1.8, 8.0 Hz, 1H), 7.41–7.39 (m, 2H), 7.34–7.30 (m, 1H). ^13^C NMR (100 MHz, CDCl_3_): δ 152.6, 150.1, 149.6, 138.9, 130.8, 125.7, 123.3, 119.5, 90.5, 89.9.

### 3.2 Structure Solution

X-ray data was collected on a Rigaku XtaLAB Synergy diffractometer using Cu Kα radiation (λ = 1.54184 Å) with a HyPix detector. Crystals were immersed in Paratone oil, and a suitable specimen was placed on a MiTeGen mount. Crystals were kept at 100.00(1) K during data collection. An analytical numerical absorption correction was applied within CrysAlisPro^41^ using a multifaceted crystal model based on expressions derived by Clark and Reid.^42^ The structures were solved in Olex2^43^ with the SHELXT^44^ structure solution program using Intrinsic Phasing and refined with the olex2.refine^45^ refinement package using Gauss–Newton minimization. The structure of the 2:2 cocrystal formed between **(8)** and **(9)** included a disordered dichloromethane that was resolved into two major components with a ratio 0.6:0.4 as the free variable converged to 0.398394. There was evidence for a very minor third component to the disorder. The final refinement for this structure was performed with SHELXL,^46^ using the Olex2 interface. In all structures, hydrogen atoms bound to carbon atoms were located in the difference Fourier map and were geometrically constrained using the appropriate AFIX commands.

### 3.3 Hirshfeld Surface Analysis and Intermolecular Interaction Energy Analysis

The program CrystalExplorer17^31^ was used to calculate the Hirshfeld surface as well as the intermolecular interaction energies within each crystal structure. In addition to calculating the interaction energies between pairs of molecules in the crystal, CrystalExplorer also decomposes the interaction energy into four physically motivated terms: (1) the classical electrostatic energy (*E*_elec_), (2) the polarization energy (*E*_pol_), (3) the dispersion energy (*E*_dis_), and (4) the exchange-repulsion energy (*E*_rep_).^32,33^

### 3.4 Electrostatic Potential Calculations

The molecules **1** to **4** were geometry optimized, with the constraint that the aromatic rings be coplanar with the N and I atoms cis relative to the pyridyl–benzene axis, using the Spartan’20 molecular modeling program with DFT at the B3LYP/6-311++G** level. The corresponding molecular electrostatic potential energy surface was calculated with an isovalue of 0.2 e/au^3^.^34^

### 3.5 Void Space Determinations

Each of the crystal structures was evaluated for void space using the program Mercury^29^ with a 1.2 Å probe radius and 0.2 Å grid spacing.

## 4 Conclusions

We have demonstrated the versatility of iodoethynylpyridine halogen bonding in the formation of discrete parallelogram-shaped self-complementary dimers and parallelogram-shaped macrocycles within both 1:1 and 2:2 cocrystals. This work supplements our earlier reports of self-complementary halogen-bonded dimers and other syntheses of supramolecular systems through cocrystallization. Our future work will further explore the principles established herein for the preparation of solid, halogen-bonded, supramolecular polygons with accessible void spaces.

## Supporting Information Available

Tabulated crystallographic data for structures **1**–**4** and cocrystals **6**–**7** and **8**–**9**; element-by-element analysis of the Hirshfeld surface of **1**–**4**; figures and tables of intermolecular interaction energies for **1**, **2**, and **3**; and figure showing void space within the structure of cocrystal **6**−**7** (PDF)
