# Supplementary material for: Halogen-Bonded Supramolecular Parallelograms: From Self-Complementary Iodoalkyne Halogen-Bonded Dimers to 1:1 and 2:2 Iodoalkyne Halogen-Bonded Cocrystals
Source: Cryst Growth Des. 2024 Feb 9;24(4):1674–81. doi: 10.1021/acs.cgd.3c01325 (PMC10885002; doi:10.1021/acs.cgd.3c01325)
Supplement: Supplementary file 1 — cg3c01325_si_001.pdf [file cg3c01325_si_001.pdf]

Supporting Information

Halogen bonded supramolecular parallelograms: From self-complementary iodoalkyne halogen bonded dimers to 1:1 and 2:2 iodoalkyne halogen bonded cocrystals

Eric Bosch[1*], Erin Speetzen[2] and Nathan P. Bowling[2]

[1]*Department of Chemistry and Biochemistry, Missouri State University, 901 South National Avenue, Springfield, Missouri, 65897, USA*

[2]*Department of Chemistry, University of Wisconsin-Stevens Point, 2001 South Avenue, Stevens Point, WI, 54481, USA*

[*] Correspondence email: ericbosch@missouristate.edu

Table of Contents

Table S1. Crystallographic data for **1** – **4** …………………………………………………… 2

Table S2. Hirshfeld surface analysis fingerprint analysis for **1** – **4** ………………………….. 3

Table S3. Interaction energies for molecules within 3.8 Å of **1**……………………………...….. 3

Figure S1. Figure of 5 strongest interacting molecules within 3.8 Å of **2**……………………. 4

Table S4. Interaction energies for molecules within 3.8 Å of **2**……………………………...….. 4

Figure S2. Figure of 5 strongest interacting molecules within 3.8 Å of **3**……………………. 5

Table S5. Interaction energies for molecules within 3.8 Å of **2**……………………………...….. 5

Table S6. Crystallographic data for cocrystals **6-7** and **8-9** ………………………..………. 6

Figure S3. Visualization of the void space within the unit cell of cocrystal **6-7**………………. 7

Table S7. Halogen bond distances and angles for all structures……………………………….. 8

Table S1. Crystallographic data for iodoalkynyl pyridines forming self-complementary dimers.[a]

	**(1)**	**(2)**	**(3)**	**(4)**
Crystal data				
Chemical formula	$C_{15}H_8IN$	$C_{15}H_8IN$	$C_{15}H_6F_2IN$	$C_{17}H_{12}IN$
M_r	329.14	329.14	365.12	357.20
Crystal system, space group	Monoclinic, $P2_1/n$	Monoclinic, $P2_1/n$	Monoclinic, $P2_1/n$	Triclinic, P-1
Temperature (K)	100	100	100	100
a, b, c (Å)	9.5916 (1), 4.8394 (1), 26.7002 (4)	9.7625 (1), 4.6880 (1), 26.9264 (2)	4.7375(1), 9.9599 (1), 27.4801 (4)	8.2005(3), 9.5231(4), 1 0.0332(5)
α, β, γ (°)	90, 93.308(1), 90	90, 95.443(1), 90	90, 94.878(1), 90	92.079(4), 100.627 (4), 110.49 (4)
V (Å^3)	1237.29(3)	1226.77(3)	1291.95(4)	717.01(6)
Z	4	4	4	2
Radiation type	Cu $K\alpha$	Cu $K\alpha$	Cu $K\alpha$	Mo $K\alpha$
μ (mm^{-1})	20.12	20.29	19.58	2.22
Crystal size (mm)	0.23 × 0.11 × 0.08	0.19 × 0.13 × 0.07	0.26 × 0.07 × 0.05	0.16 × 0.06 × 0.04
Data collection				
T_{min}, T_{max}	0.104, 0.346	0.115, 0.382	0.059, 0.478	0.785, 0.933
No. of measured, independent and observed [$I \geq 2u(I)$] reflections	12063, 2480, 2380	11911, 2447, 2380	23571, 2668, 2617	18097, 3472, 3058
R_{int}(sin θ/λ)$_{max}$ (Å^{-1})	0.029	0.034	0.044	0.045
Refinement				
$R[F^2 > 2\sigma(F^2)]$, $wR(F^2)$, S	0.021, 0.055, 1.04	0.019, 0.053, 1.04	0.022, 0.058, 1.04	0.033, 0.077, 1.03
No. of reflections	2480	2447	2668	3472
No. of parameters	154	155	173	174
$\Delta\rho_{max}$, $\Delta\rho_{min}$ (e Å^{-3})	0.65, −0.99	0.52, −0.73	0.72, −0.80	2.37, −1.19
CCDC refcode	2303945	2303946	2303947	2303948

Data collected at 100 K on a XtaLAB Synergy Dualflex, HyPix diffractometer with Cu $K\alpha$ radiation for **1**, **2** and **4** and Mo $K\alpha$ for **3**.

Table S2. Hirshfeld analysis of self-complementary dimers **1** – **4** with element-by-element delineation of the % contribution. In all cases reciprocal contacts are included.

Cpd	**N---I**	**C---C**	**C---H**	**H---H**	**C---I**	**C---N**	**N---H**	**I---H**	**F---H**	**F---C**
**1**	5.3	10.6	34.2	27.2	4.4	2.2	2.1	13.2	-	-
**2**	4.5	15.1	28.4	27.2	4.4	1.5	3.3	15.4	-	-
**3**	5.1	12.9	22.6	10.3	4.0	2.0	2.1	13.6	19.3	6.0[b]
**4**	3.1	12.2	25.6	36.2	1.5	0.5	4.0	17.0	-	-

[b]Also 1.3% F---F contacts.

Table S3. Interaction energies of 4 unique molecules within 3.8 Å of molecule **(1)** in Fig. 5.

Colour and code	R	E_{ele}	E_{pol}	E_{dis}	E_{rep}	E_{tot}
Green, XB2	7.10	-146.3	-18.1	-22.0	212.2	-56.2
Light blue, OPI	4.84	-19.2	-2.3	-62.6	62.3	-38.1
Dark blue, BCHP	9.70	-13.2	-1.1	-21.4	23.7	-18.8
Pink, OF	5.41	-13.9	-0.6	-27.1	36.1	-16.4

R is the distance between molecular centroids in Å and all energies are given in kJ/mol. Scale factors for benchmarked energy models see Mackenzie (2017).

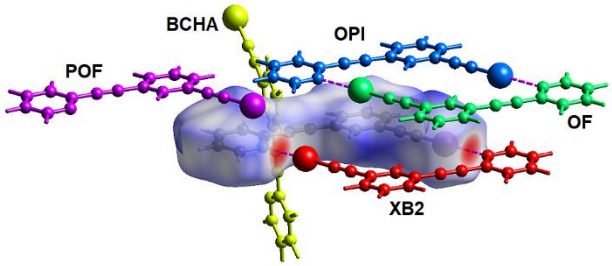

Figure S1. View of the 5 unique molecules within 3.8 Å of the central grey molecule **2** that have the strongest intermolecular interaction energy with central molecule **2**. Color coded as: Red = halogen bonded, XB2; blue = offset π-stacked, OPI; green = offset, OF; chartreuse = bifurcated C-H---alkyne, BCHA; pink = parallel offset, POF.

Table S4. Interaction energies of 5 unique molecules within 3.8 Å of molecule **2** in Fig. S1.

Colour and code (Fig. S1)	R	E_{ele}	E_{pol}	E_{dis}	E_{rep}	E_{tot}
Red, XB2	4.33	-129.1	-16.5	-36.1	191.0	-62.1
Blue, OPI	4.69	-18.1	-2.7	-58.2	58.0	-36.0
Chartreuse, BCHA	9.88	-14.5	-1.1	-22.0	25.6	-19.5
Green, OF	6.17	-8.2	-0.4	-21.3	20.1	-15.0
Pink, POF	10.83	-8.5	-1.0	-21.3	22.1	-14.5

R is the distance between molecular centroids in Å and all energies are given in kJ/mol. Scale factors for benchmarked energy models see Mackenzie (2017).

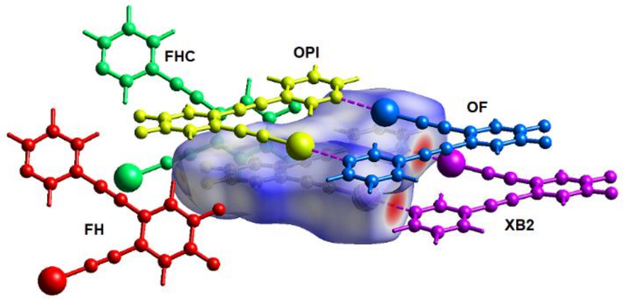

Figure S2. View of the 5 unique molecules within 3.8 Å of the central grey molecule **(3)** that have the strongest intermolecular interaction energy with central molecule **(3)**. Colour coded as: Pink = halogen bonded, XB2; chartreuse = offset π-stacked, OPI; blue = offset, OF; FHC = C-F—H and C, FHC; red = C-F---H, FH. The I---N halogen bonds shown as dashed lines.

Table S5. Interaction energies of 5 unique molecules within 3.8 Å of molecule **(3).**

Colour and code (Fig. S2)	R	E_{ele}	E_{pol}	E_{dis}	E_{rep}	E_{tot}
Pink, XB2	8.33	-144.5	-17.7	-21.7	206.1	-57.4
Chartreuse, OPI	4.74	-27.6	-1.4	-71.6	74.1	-46.8
Green, FHC	9.33	-8.0	-1.3	-22.6	22.5	-15.2
Red, FH	11.73	-7.0	-0.6	-10.6	9.4	-11.2
Blue, OF	6.44	-11.6	-0.4	-25.3	29.7	-16.2

R is the distance between molecular centroids in Å and all energies are given in kJ/mol.

Scale factors for benchmarked energy models see Mackenzie (2017).

Table S6. Crystallographic data for cocrystals.[a]

	**(6-7)**	**(8-9)**
Crystal data		
Chemical formula	$C_{38}H_{24}I_2N_2O_2$	$C_{22}H_{10}F_2I_2N_2 \cdot CH_2Cl_2$
M_r	794.44	679.08
Crystal system, space group	Monoclinic, $P2_1/n$	Monoclinic, $I2/a$
Temperature (K)	100	100
a, *b*, *c* (Å)	19.5889(2), 8.2416(1), 20.7117(2)	27.635(4), 5.2732(1), 32.2601(4)
α, β, γ (°)	90, 102.977(1), 90	90, 94.221(1), 90
V (Å^3)	3258.38 (6)	1226.77 (3)
Z	4	8
Radiation type	Cu $K\alpha$	Cu $K\alpha$
μ (mm^{-1})	15.44	23.43
Crystal size (mm)	0.24 × 0.16 × 0.12	0.11 × 0.07 × 0.04
Data collection		
T_{min}, T_{max}	0.091, 0.636	0.198, 0.535
No. of measured, independent and observed [$I \geq 2u(I)$] reflections	43875, 6536, 6078	26880, 4994, 4399
R_{int}(sin θ/λ)$_{max}$ (Å^{-1})	0.045	0.055
Refinement		
$R[F^2 > 2\sigma(F^2)]$, $wR(F^2)$, S	0.030, 0.080, 1.05	0.042, 0.118, 1.02
No. of reflections	6536	4994
No. of parameters	399	298
$\Delta\rho_{max}$, $\Delta\rho_{min}$ (e Å^{-3})	1.09, −0.89	1.98, −1.20
CCDC refcode	2304143	2304144

[a] Data collected at 100(2) K on a XtaLAB Synergy Dualflex, HyPix diffractometer with Cu $K\alpha$ radiation.

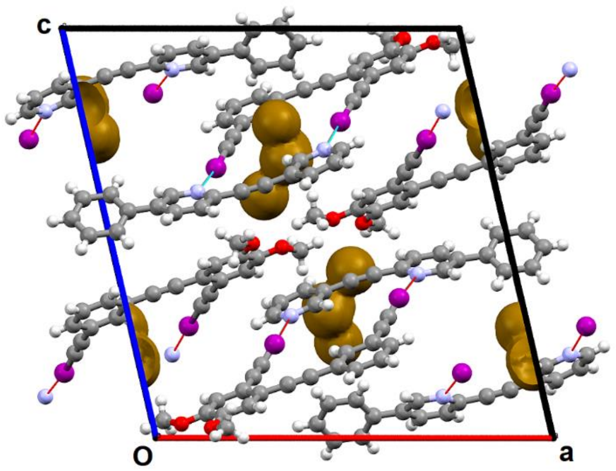

Figure S3. Visualization of the void space, shown in yellow, within the unit cell of cocrystal **6-7** viewed along the *b* axis.

Table S7. Halogen bond distances and angles for all structures in this manuscript.

Compound	**1**	**2**	**3**	**4**	**6-7**	**8-9**
I---N dist., Å	2.769(2)	2.8424(19)	2.780(2)	2.967(3)	2.804(3), 2.823(3)	2.733(4), 2.776(5)
C-I---N ang., º	175.67(8)	175.55(8)	176.5(9)	175.78(11)	178.00(11), 174.85(12)	174.28(19), 176.57(18)

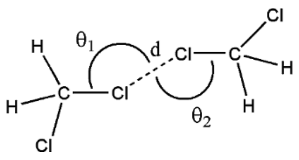

Figure S4. Distance and angle criteria for Cambridge Database search: separation, d, from 2.6 to 3.4 Å with θ_1 and θ_2 constrained between 120 and 180º and $\theta_1 = \theta_2$. Search for single crystal structures of organics with no errors, non-disordered and no ions.
